# Sodium zirconium cyclosilicate versus sodium polystyrene sulfonate for treatment of hyperkalemia in hemodialysis patients: a randomized clinical trial

**DOI:** 10.1186/s12882-025-04129-9

**Published:** 2025-05-06

**Authors:** Mohamed Mamdouh Elsayed, Marwa Ahmed Abdelrahman, Abdelrazik Mohamed Sorour, Islam Ghanem Rizk, Mohamed Aly Abdelhalim Hassab

**Affiliations:** 1 Nephrology and Internal Medicine Department, Faculty of Medicine, Alexandria University, Alkhartoom Square, El Azareeta, Alexandria, 21131 Egypt; 2 Nephrology Department, Alexandria Petroleum Hospital, Alexandria, Egypt; 3 Nephrology and Internal Medicine Department, Medical Research Institute, Alexandria University, Alexandria, Egypt

**Keywords:** Sodium zirconium cyclosilicate, Sodium polystyrene sulfonate, Hyperkalemia, Hemodialysis

## Abstract

**Background:**

Hyperkalemia is a frequent life-threatening condition in hemodialysis (HD) patients. Data comparing the usage of various K + binders in HD patients is still scarce. This study aimed to compare the efficacy and safety of Sodium zirconium cyclosilicate (SZC) and sodium polystyrene sulfonate (SPS) for treatment of hyperkalemia in HD patients.

**Methods:**

This prospective, double-blinded, randomized multicenter clinical trial enrolled 120 HD patients with predialysis serum potassium > 5 mmol/L. Patients were randomized to receive SZC (5 g, 3 times/wk on non-dialysis days, 15 gm/wk) or SPS (15 g, 3 times/wk on non-dialysis days, 45 gm/wk) for 8 weeks. The change in serum potassium through the 8 weeks of the study was our primary outcome.

**Results:**

Serum potassium significantly decreased in both groups compared to baseline values from the first week till the end of the study with *p* value of < 0.001 and < 0.001 respectively. Serum K levels in the SZC group were significantly lower (achieved normokalemia after 2 weeks) than K levels in the SPS group (achieved normokalemia after 6 weeks) through the study period (*p* < 0.001). Rescue therapy for hyperkalemia was less frequent in the SZC group (3.3%) than the SPS group (6.6%) (*p* = 0.678). Gastrointestinal side effects were non significantly fewer with SZC (5%) compared to SPS (11.6%). However, SPS was less palatable (*p* < 0.001).

**Conclusions:**

When compared to SPS treatment, SZC was associated with a more rapid and efficacious resolution of hyperkalemia with potentially a better safety profile and palatability among HD patients.

**Clinical trials registration:**

ClinicalTrials.gov Identifier: NCT06029179. First registration date: 9/01/2023.

## Background

Currently, around three million patients worldwide are end-stage renal disease (ESRD) and dependent on renal replacement therapy, mainly maintenance hemodialysis (HD) with forecasts estimating between five and ten million by 2030 [1]. Patients with ESRD, especially the ones undergoing HD are at high risk of developing hyperkalemia, which is usually defined as serum potassium (K+) concentrations > 5.0 mmol/l, even with thrice-weekly HD sessions [2]. Hyperkalemia can result in sudden cardiac death if not urgently controlled [3].

The main approaches for managing the hyperkalemia in ESRD patients include dietary K + restriction, additional dialysis sessions, reducing the dialysate K + concentration, and the avoidance of drugs that increase serum K + levels. The gastrointestinal (GI) system eliminates approximately 10% of daily K + intake. Potassium transport along the colon is different, with the proximal colon revealing net secretion and the distal colon revealing net absorption [4]. In addition, recent data show an increase in colonic K + secretion in dialysis patients [5]. Therefore, to avoid pre-dialysis hyperkalemia, a long-term therapy using drugs promoting intestinal K + elimination is applied, for which the GI tract offers a potentially major novel avenue for K + excretion [4].

Until recently, the only K+-binding agents used to reverse hyperkalemia were sodium polystyrene sulfonate (SPS) and calcium polystyrene sulfonate (CPS). SPS works in the colon as a cation-exchange resin, binding K + ions nonspecifically in exchange for sodium in a K + concentration-dependent manner [6]. In small doses, SPS can be used to treat hyperkalemia in patients receiving continuous HD [7]. It has not proved effective in large prospective trials, and there is inadequate long-term evidence of beneficial effects. Additionally, this K+-binding drug has been associated with GI discomfort and disorders [8, 9]. The sodium zirconium cyclosilicate (SZC) and patiromer are additional K+-binders that were recently authorized for the management of hyperkalemia [10]. Unlike SPS and patiromer that work mainly in the colon, published data suggest that SZC acts across a larger surface area in the GI tract including the intestine (duodenum and jejunum). Furthermore, SZC shows very selective pH-independent binding of K+ [11]. SZC is capable of keeping predialysis K + values within 4.0 to 5.0 mmol/l range [12]. So far, there is insufficient comparative data on different K + binders in HD patients.

We conducted this study to be the first to compare the efficacy and safety of SZC and SPS for the treatment of hyperkalemia in HD patients.

## Materials and methods

### Study participants and design

This study is a prospective, double-blinded, multicenter randomized clinical trial which included 120 patients from various dialysis facilities in Alexandria. We enrolled ESRD patients with predialysis serum potassium level > 5 mEq/L, older than 18 years, and treated by regular HD (three times a week, by high flux dialyzers, four hours per HD session for more than three months). Dialysate K concentration was fixed for all patients at 2 mmol/L. Patients were randomly assigned using the block randomisation technique to receive sodium zirconium cyclosilicate (SZC) 5 g, 3 times/wk on non-dialysis days (15 gm/week) or sodium polystyrene sulfonate (SPS) 15 g, 3 times/wk on non-dialysis days (45 gm/week) for 8 weeks. Medical insurance covered the costs of both drugs as our patients were already hyperkalemic and need the treatment. The choice of the anti-hyperkalemic agent was up to the physician decision. Each patient was given a code for identification, and allocation concealment was ensured by using the sealed closed envelop randomisation technique. All patients received standard dietary advice to optimize their nutritional intake in accordance with the Kidney Disease Outcomes Quality Initiative (KDOQI) guidelines and were closely monitored during the trial period. We used the 3-day food record to assess the dietary intake. In patients with residual urine output (UOP), use of diuretics was not allowed during study period to avoid any influence on K removal. We excluded patients with GI diseases (bleeding, constipation, history of endoscopy, malabsorption, diarrhea in the past month or chronic diarrhea, perforation, necrosis, ischemic colitis, GIT surgery), use of chronic laxatives, myocardial infarction (MI), acute coronary syndrome (ACS), pregnancy, breast feeding, seizure, stroke, or thromboembolic event within 8 weeks before study. We also excluded those who received within 2 weeks before the study any medications to control hyperkalemia. The trial was registered on Clinicaltrials.gov (NCT06029179) (9/01/2023) and was conducted in accordance with the CONSORT 2010 statement.

### Methods

Each patient had a comprehensive medical history with focus on demographic data, etiology of ESRD, the vintage of HD, comorbidities, and medication history. Thorough physical examination was done with stress on interdialytic weight gain (IDWG), blood pressure, fluid overload. Laboratory investigations included serum potassium measurement using Quicklyte integrated multisensor on Dimension Exl 200 (Siemens healthineers, USA), serum sodium, complete blood count, serum phosphorus, calcium, PTH, creatinine, urea, albumin, triglycerides.

### Study outcomes

Primary outcome was the change in serum potassium through the 8 weeks of the study. Serum K was assessed at baseline, 1st and 2nd week, and then every 2 weeks after the long interdialytic interval. Secondary outcomes included change in interdialytic weight, change in blood pressure, laboratory parameters assessment, need for rescue therapy for hyperkalemia (agents other than SZC and SPS were used when serum K level > 6 mmol/L, for example beta-adrenergic agonists, insulin/glucose). Patients were monitored for AEs and any serious AEs during study and for 8 weeks after study end.

### Statistical analysis

IBM SPSS software package version 20.0 was used to analyze the data that were fed into the computer (IBM Corporation, Armonk, NY, 2011). Numbers and percentages were used to represent categorical data. Two groups were compared using the Chi-square test. Alternatively, when more than 20% of the cells had an anticipated count below 5, the Fisher Exact test was used. The Shapiro-Wilk test was applied to determine the normality of continuous data. The range (minimum and maximum), mean, standard deviation, median, and interquartile range (IQR) were used to express the quantitative data. Two groups were compared using the student t-test for quantitative variables that were normally distributed. While two periods were compared using Paired t-test. On the other hand, for non-normally distributed quantitative variables Mann Whitney test was used to compare two groups. While two periods were compared using Wilcoxon signed ranks test. The significance of the results obtained was judged at the 5% level. The Power Analysis and Sample Size Software (PASS 2020) “NCSS, LLC. Kaysville, Utah, USA, ncss.com/software/pass” was applied to determine the sample size. The minimal total hypothesized sample size of 120 eligible patients (60 per group) is needed to compare the effects of SZC versus SPS for treatment of hyperkalemia in patients undergoing regular HD; taking into consideration 95% level of confidence, effect size of 25%, and power of 80% using Chi square- test [12].

## Results

### Baseline characteristics of patients

**Fig. 1:**
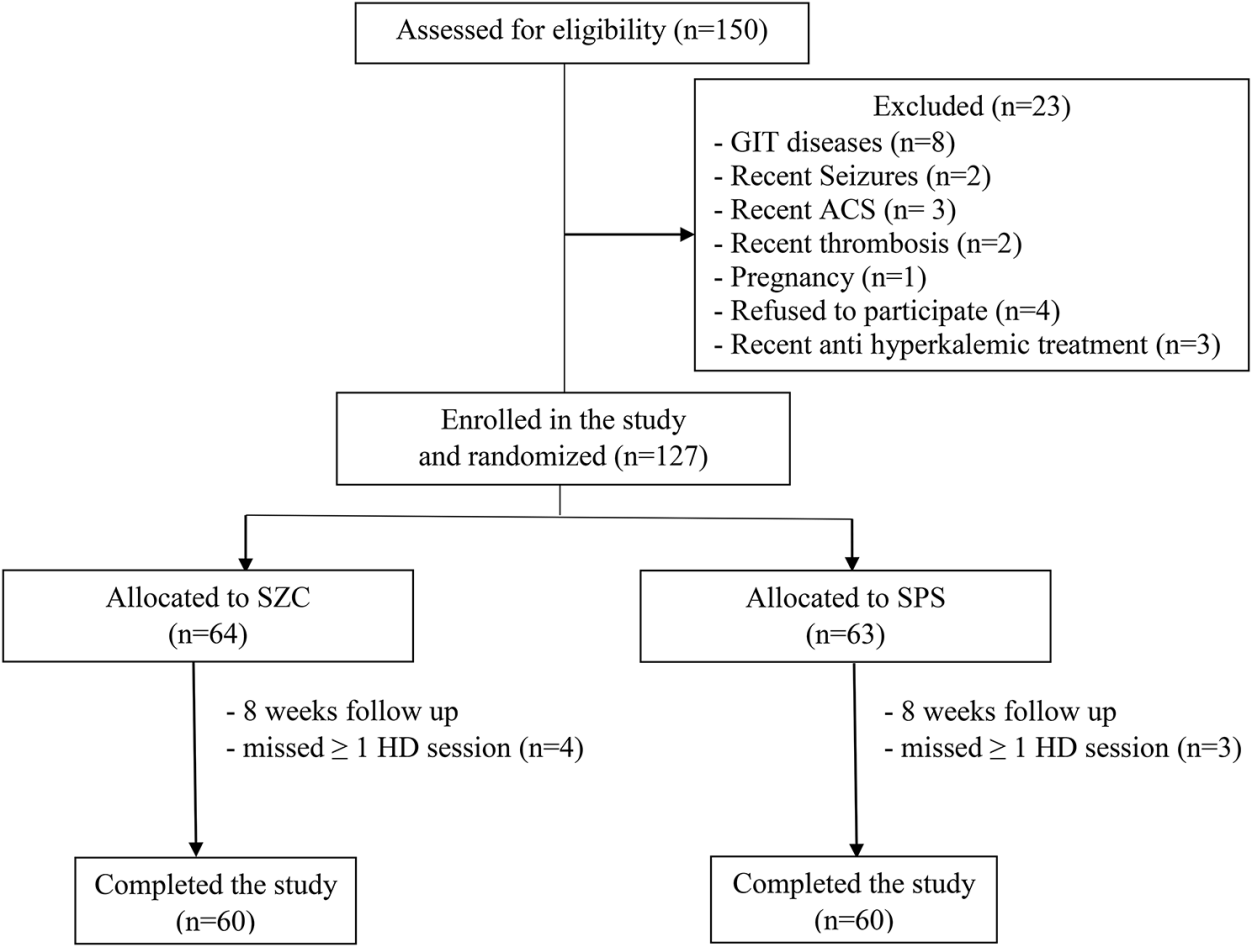
Patient flow chart

A total of 150 HD patients were evaluated for participation in the study. Among these, 19 were excluded, 7 patients missed more than ≥ 1 HD session, and 4 refused to participate. In total, 120 HD patients completed the study. Sixty patients received SZC 15 gm/week, and the other 60 patients received SPS 45 gm/week for 8 weeks (Fig. 1). There was no statistically significant difference between both groups regarding age, sex, comorbidities, body mass index (BMI), duration of HD, vascular access, cause of ESRD, dialysis related data, dialysis adequacy (Kt/V), presence of residual UOP and various laboratory parameters. Table 1 shows the clinical features of the patients.

**Table 1:** Baseline characteristics of patients

	SZC group (*n* = 60)	SPS group (*n* = 60)	*P*
**Age** (years)	47.4 ± 10.4	51.6 ± 13.0	0.083
**Sex**			
Male	33 (55%)	31 (51.6%)	0.841
Female	27 (45%)	29 (48.3%)	
**BMI** (kg/m^2^)	27 ± 4.5	27.8 ± 5.9	0.449
**Duration of HD** (years)	7 ± 4.6	6.6 ± 6.1	0.412
**Cause of ESRD**			
Hypertension	21 (35%)	27 (45%)	
DM	12 (20%)	15 (25%)	
Chronic pyelonephritis	6 (10%)	3 (5%)	
Glomerulonephritis (GN)	8 (13.3%)	5 (8.3%)	
ADPCKD	6 (10%)	4 (6.6%)	
Others	7 (11.6%)	6 (10%)	
**Potassium raising medications**			
ACEIs/ARBs use	5 (8.3%)	5 (8.3%)	1.000
MRAs use	1 (1.6%)	1 (1.6%)	1.000
**Vascular access**			
AVF	48 (80%)	54 (90%)	0.102
Catheter	12 (20%)	6 (10%)	
**HD prescription**			
Blood flow rate (ml/min) (QB)	318.2 ± 41.0	307.2 ± 34.1	0.146
Dialysate flow (ml/hr) (QD)	594.4 ± 103.5	577.2 ± 94.8	0.391
UF volume (L/session)	3.7 (2.5 − 4.7)	3.25 (3 − 4)	0.147
**Presence of residual UOP**			
Yes	35 (58.3%)	33 (55%)	0.732
No	25 (41.7%)	27 (45%)	
**Total cholesterol** (mg/dl)	179.4 ± 25.8	182.2 ± 36.0	0.656
**Serum triglycerides** (mg/dl)	131 (107–178)	133.5 (115–150)	0.603
**Hemoglobin** (g/dl)	9.6 ± 1.8	9.6 ± 1.7	0.857
**Albumin** (g/dl)	3.85 ± 0.3	3.76 ± 0.4	0.218
**Serum electrolytes**			
Calcium (mg/dl)	8.9 ± 1.1	9.28 ± 0.9	0.078
Phosphorus (mg/dl)	5.8 (4.9–7)	6 (5.3–6.6)	0.779
Sodium (mEq/L)	136.1 ± 15.8	136.1 ± 3.8	0.993
Potassium (mEq/L)	5.6 ± 0.2	5.6 ± 0.3	0.892
**Serum PTH** (pg/ml)	301 (132.6–494)	456 (179–822.5)	0.180
**CRP** (mg/l)	5.5 (4–13)	4.9 (3.2–9.1)	0.273
**Kt/V**	1.42 ± 0.3	1.40 ± 0.2	0.690
**URR**	69.7 ± 9.6	69.5 ± 8.6	0.926

*p*: p value for comparing between the two groups

Normally quantitative data were expressed as Mean ± standard deviation (SD) while non-normally quantitative data were expressed as Median with interquartile ranges (IQR), or absolute numbers as appropriate

ACEIs: angiotensin converting enzyme inhibitors, ADPCKD: autosomal dominant polycystic kidney disease, ARBs: angiotensin receptor blockers, AVF: arteriovenous fistula, BMI: body mass index, CRP: C reactive protein, DM: diabetes mellitus, ESRD: end stage renal disease, HD: hemodialysis, Kt/V: measuring dialysis adequacy, MRAs: mineralocorticoid antagonists, PTH: parathyroid hormone, UF: ultrafiltration, UOP: urine output, URR: urea reduction ratio

### Effect of SZC and SPS on serum K

**Fig. 2:**
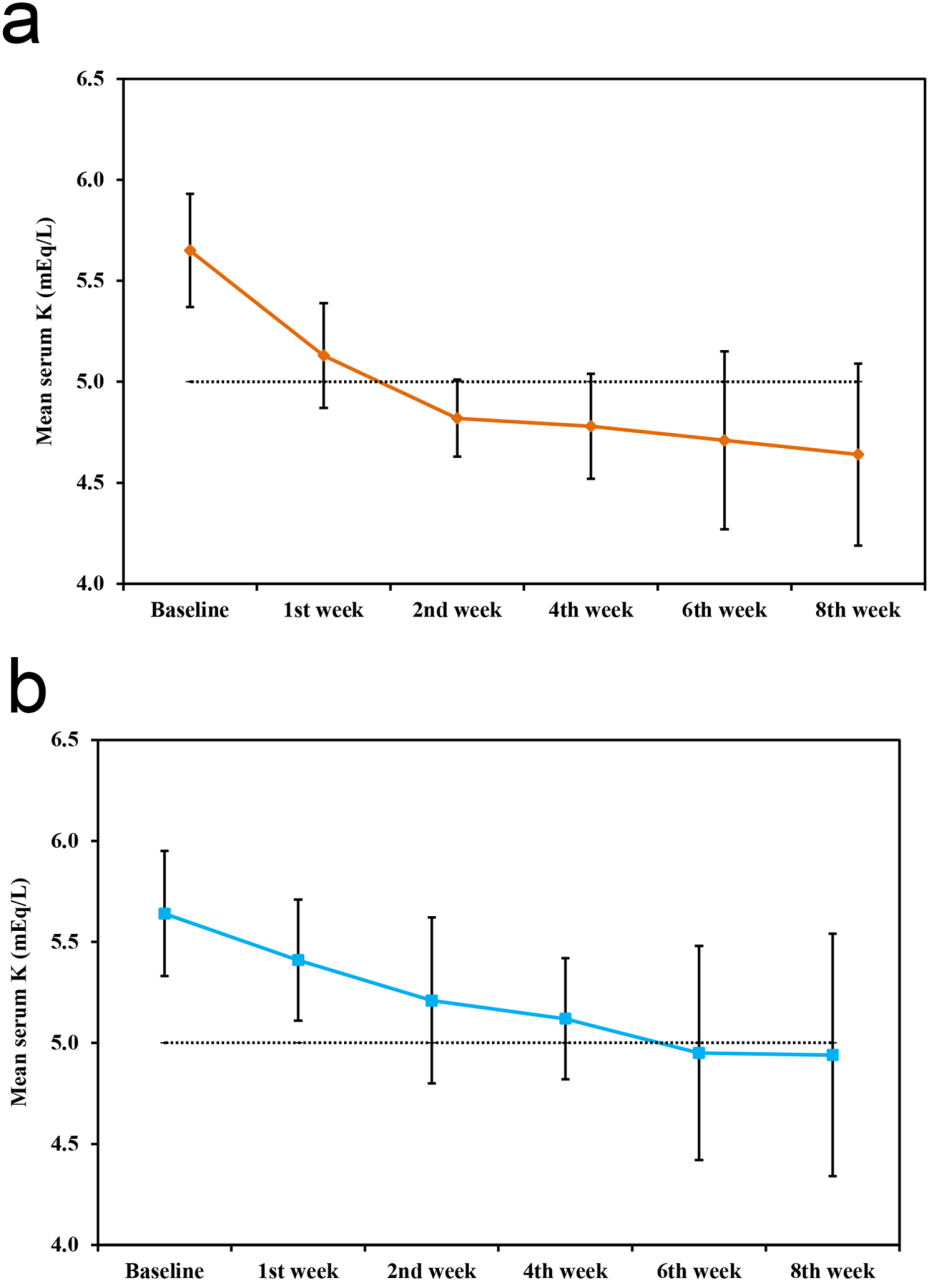
Serum potassium during study period in both groups. **a** Changes in serum potassium in SZC group. **b** Changes in serum potassium in SPS group

At baseline, there was no significant difference between both groups regarding serum K (*p* = 0.892). After initiating treatment, serum K dropped significantly in both groups compared to baseline values from the first week till the end of the study with *p* value of < 0.001 and < 0.001 respectively. Serum K levels in the SZC group were significantly lower than K levels in the SPS group through the study period (*p* < 0.001). The mean serum K in the SZC group reached normokalaemia (< 5 mmol/L) after 2 weeks of treatment. However, the mean serum K reached normokalaemia only after 6 weeks of SPS treatment (Fig. 2).

### Secondary outcomes of the study

Interdialytic weight gain (IDWG), blood pressure measurements, serum sodium, calcium, phosphorus and albumin levels did not significantly differ between both groups at baseline and at study end. Rescue therapy for hyperkalemia was needed in 4 patients in the SPS group, and in 2 patients in the SZC group without a significant difference (*p* = 0.678) (Table 2).

**Table 2:** Secondary outcomes of the study

	SZC group (*n* = 60)				SPS group (*n* = 60)					Comparison bet. gps. at baseline	Comparison bet. gps. at week 8
	Baseline	Week 8	*P*_0_		Baseline	Week 8		*p*_0_		*P*_1_	*P*_2_
**Interdialytic weight gain** (kg)	3.0 (2.0–4.2)	3.0 (2.0–4.0)	0.205		3.0 (2.5–3.5)	3.0 (2.0–4.0)		0.624		0.997	0.793
**BP** (mmHg)											
- Systolic BP	134.4 ± 25.1	136.4 ± 16.7	0.636		136.3 ± 18.4	138.5 ± 17.3		0.506		0.664	0.540
- Diastolic BP	79.3 ± 14.6	81.2 ± 9.6	0.369		80.7 ± 10.9	82.3 ± 9.8		0.415		0.590	0.552
**Need for rescue therapy** (No)	-	2 (3.3%)	NA		-	4 (6.6%)		NA		-	0.678
**Albumin** (g/dl)	3.8 ± 0.3	3.8 ± 0.4	0.634		3.7 ± 0.4	3.7 ± 0.5		0.765		0.218	0.387
**Serum electrolytes**											
Sodium (mEq/L)	136.1 ± 15.8	138.4 ± 4.1	0.324		136.1 ± 3.8	137.5 ± 3.7		0.074		0.993	0.266
Calcium (mg/dl)	8.9 ± 1.1	9.1 ± 1.3	0.357		9.2 ± 0.9	9.2 ± 1.3		0.892		0.078	0.604
Phosphorus (mg/dl)	5.8 (4.9–7.0)	5.2 (3.7 − 7.3)	0.440		6.0 (5.3–6.6)		5.6 (4.3 − 7.4)	0.881		0.779	0.387

Normally quantitative data were expressed as Mean ± standard deviation (SD) while non-normally quantitative data were expressed as Median with interquartile ranges (IQR), or absolute numbers as appropriate

^*t*^*p*_*0*_: p value for comparing between baseline and week 8 in each group; *p*_*1*_: p value for for comparison bet. groups at baseline; *p*_*2*_: p value for for comparison bet. groups at week 8; *: Statistically significant at *p* ≤ 0.05; BP: blood pressure

### Safety and adverse events

A summary of the adverse events among the patients studied is shown in Table 3. Serious adverse events occurred in two patients in the SZC group (MI, catheter related blood stream infection), and in three patients in the SPS group (shunt thrombosis, pulmonary edema and severe chest infection) (*p* = 1.000). GIT side effects (diarrhea, constipation, nausea) were reported in 3 patients in the SZC group, and in 7 patients in the SPS group leading to drug discontinuation in one patient. SZC was significantly more palatable than the SPS (*p* < 0.001).

**Table 3:** Adverse events in study groups

	SZC group (n = 60)	SPS group (n = 60)	Comparison bet. groups *p*
**Serious AE**	2 (3.3%)	3 (5%)	1.000
**AE causing drug discontinuation**	0	1 (1.6%)	1.000
**GIT AEs**			
- Diarrhea	1 (1.6%)	2 (3.3%)	1.000
- Constipation	2 (3.3%)	3 (5%)	1.000
- Nausea	0 (0.0)	2 (3.3%)	0.495
**Headache**	1 (1.6%)	1 (1.6%)	1.000
**Hypokalemia**	0	0	-
**Poor palatability**	2 (3.3%)	17 (28.3%)	< 0.001^*^

Data were expressed as absolute numbers as appropriate

AE: adverse events, GIT: gastrointestinal tract

*: Statistically significant at p ≤ 0.05

## Discussion

Hyperkalemia in HD patients poses significant risks, requiring effective and well-tolerated treatments. While traditional agents like SPS have limitations, SZC offers a novel approach with faster action and fewer side effects. This study is considered the first prospective trial to evaluate in the same study the efficacy and safety of these agents to enhance hyperkalemia management in this vulnerable population (HD) [13–15]. This study compares the efficacy and safety of these two agents, shedding light on innovative strategies to optimize hyperkalemia management and improve outcomes for patients reliant on renal replacement therapy.

In the present study, significant reduction in serum potassium levels was observed in both groups during the study period. SZC achieved normokalemia (< 5 mmol/L) faster (by 2 weeks) compared to SPS (by 6 weeks). Mean serum potassium levels were significantly lower in the SZC group compared to the SPS group throughout the study (*p* < 0.05).

In accordance, Fu et al. included 73 CKD (non-dialysis) patients to test the efficacy and safety of SZC and CPS in controlling potassium in patients with acute and severe hyperkalemia and found a significant reduction in serum potassium in both groups, with higher potassium reduction in the SZC group. Also, the SZC group showed a significantly higher control rate for hyperkalemia than in the CPS group [16]. Also, Thai et al. included 46 patients with 17 in the SZC group and 29 in the SPS group. Potassium normalization was attained in 16 (94%) in the SZC group and 27 (93%) in the SPS group. Statistically, neither SZC nor SPS was more efficacious; however, the quicker onset of SZC could provide a clinically meaningful difference in the treatment of acute hyperkalemia [17]. Furthermore, Yoo et al. included 260 hyperkalemic patients who were given either a single dosage of SZC or one or more SPS doses and found a similar initial serum potassium in both groups (5.6 ± 0.4). The absolute reduction in serum potassium was − 0.88 mEq/L and − 0.75 mEq/L with SZC and SPS, respectively. When compared to SPS, the SZC “once” regimen showed no inferiority (*P* < 0.0001) [18].

In contrast, Hasara et al. who assessed the SPS versus SZC in managing hyperkalemia in the ED reported a − 1.1 mEq/L reduction in serum K in both groups in the first measurement following administration of drugs indicating no superiority for one on other [19]. Also, Sullivan et al. in their study revealed a mean drop in serum potassium of 0.96 mEq/L with SPS and 0.78 mEq/L with SZC within 24 h following binder administration (*P* < 0.0001). Although the SPS resulted in a statistically higher drop in serum potassium, there was a considerable variability in the doses applied limiting the comparison ability of specific doses [20]. Furthermore, Lewis et al. found that the SZC 10 g, SZC (3 doses of 10 g), and SPS 15–30 g resulted in a potassium reduction of 0.70 mmol/L, 0.78 mmol/L, and 0.99 mmol/L within 12–30 h, respectively (*p* < 0.01). This difference may also be attributed to variability of doses used in each group [21].

In the present study, no significant differences between groups in interdialytic weight gain, blood pressure, serum sodium, calcium, phosphorus, or albumin levels. Rescue therapy was required less frequently in the SZC group (4%) than in the SPS group (8%), though not statistically significant (*p* = 0.678). Similarly, according to Fu et al., serum levels of sodium, magnesium, calcium, and phosphorus did not significantly change within 72 h following medication consumption without any reported severe adverse events [16]. SZC selectively binds potassium ions across a larger surface area in the GI in exchange for hydrogen and sodium ions. This mechanism minimizes potassium reabsorption without significant sodium overload or disruption of other electrolyte balances. Its high selectivity reduces the likelihood of interfering with calcium, phosphorus, or albumin levels [22]. While SPS works mainly in the colon by exchanging potassium for sodium ions, but its action is less selective. Despite this, the exchange process generally does not significantly affect other systemic electrolyte levels [23].

In the current study, serious adverse events occurred in 4% of SZC patients and 6% of SPS patients, with no statistical significance (*p* = 1.000). GI side effects (e.g., diarrhea, constipation, nausea) were less frequent in the SZC group (5%) compared to the SPS group (11.6%), leading to drug discontinuation in one SPS patient. Also, SZC demonstrated better tolerability, with fewer adverse events overall. Similarly, Joyce and Corpman included 246 patients; and found a similar efficacy and safety outcomes between SZC and SPS. Only the SPS group experienced the five major adverse events that were documented [24]. In a RCT by Fishbane et al., SZC was effective and well tolerated in hyperkalemic ESKD patients undergoing maintenance HD [12]. Regarding palatability, we found that SZC was significantly more palatable than SPS in our patients (*p* < 0.001). Similar findings were reported by Wheeler et al. [25].

Our study’s strength points include being the first prospective trial to compare SZC and SPS directly in HD patients in the same study. In addition, we assessed this comparison taking into consideration efficacy and safety outcomes, including serum K, BP, IDWG, laboratory parameters, and adverse events. Possible limitations of our study might be the relatively small sample size (*n* = 120), short follow-up duration (8 weeks), and longer periods would have significantly strengthened our findings. Also, we did not use different doses for both drugs which could have provided more data about the efficacy and flexibility of administration. In addition, we did not calculate the amount of K in diet (mEq/day) due to difficulty of precise estimation. Also, despite the non-significant difference between both groups regarding the presence of residual UOP, but we did not measure the amount of UOP in those patients. Additionally as illustrated above, we excluded from the study any patient with constipation, diarrhea or on chronic laxatives, but we did not take into consideration the frequency of bowel movements in the included patients which can affect K removal. Finally, our study design did not include a cross over phase which could have significantly support our findings.

## Conclusion

SZC demonstrated superior efficacy in achieving faster potassium normalization and maintaining serum potassium levels within the target range compared to SPS. Its favorable safety profile, with fewer gastrointestinal side effects, suggests that SZC may be a more effective and well-tolerated alternative for managing hyperkalemia in HD patients.

## Data Availability

The datasets used and/or analysed during the current study are available from the corresponding author on reasonable request.

## Abbreviations

SZC: Sodium zirconium cyclosilicate
SPS: Sodium polystyrene sulfonate
HD: Hemodialysis
CPS: Calcium polystyrene sulfonate
